# Using proteomics to identify host cell interaction partners for VgrG and IglJ

**DOI:** 10.1038/s41598-020-71641-3

**Published:** 2020-09-03

**Authors:** Magdalena Proksova, Helena Rehulkova, Pavel Rehulka, Claire Lays, Juraj Lenco, Jiri Stulik

**Affiliations:** 1 Department of Molecular Pathology and Biology, Faculty of Military Health Sciences, University of Defence, Trebesska1575, Hradec Kralove, Czech Republic; 2 CIRI, International Center for Infectiology Research, Inserm U1111, UMR5308, CNRS, Lyon, France; 3 Faculty of Pharmacy, Charles University, Hradec Kralove, Czech Republic

**Keywords:** Mechanisms of disease, Bacterial infection

## Abstract

*Francisella tularensis* is a highly virulent intracellular bacterium and the causative agent of tularemia. The disease is characterized by the suboptimal innate immune response and consequently by the impaired adaptive immunity. The virulence of this pathogen depends on proteins encoded by a genomic island termed the *Francisella* Pathogenicity Island (FPI). However, the precise biological roles of most of the FPI-encoded proteins remain to be clarified. In this study, we employed stable isotope labeling by amino acids in cell culture (SILAC) in combination with affinity protein purification coupled with liquid chromatography–mass spectrometry to identify potential protein-effector binding pairs for two FPI virulence effectors IglJ and VgrG. Our results may indicate that while the IglJ protein interactions primarily affect mitochondria, the VgrG interactions affect phagosome and/or autophagosome biogenesis via targeting components of the host’s exocyst complex.

## Introduction

*Francisella tularensis* is a Gram-negative, facultative intracellular bacterium responsible for the zoonotic disease tularemia. This highly virulent pathogen spreads easily through aerosol. Indeed, only a few colony forming units are necessary to cause the disease in humans. Therefore, *F. tularensis* is classified as biothreat agent category A by the U.S. Centers for Disease Control and Prevention^1,2^.

The bacterium enters, survives, and proliferates within a variety of host-cell types, such as dendritic cells, polymorphonuclear neutrophils, endothelial cells, type II alveolar lung epithelial cells, and hepatocytes^3^. Macrophages are the most important target among the phagocytic cells^4^. Soon after phagocytosis, the bacteria inhabit the *Francisella* containing phagosome, which acquires early and subsequently late endosomal markers EEA1, Lamp1, Lamp2, Rab7, and Rab5. The *Francisella* containing phagosome does not, however, mature into a phagolysosome and it fails to acquire lysosomal marker cathepsin D^5–7^. Instead of being killed, the *F. tularensis* escapes from the phagosome and replicates in host-cell cytosol. Not only does the major cellular component of the primary line of defense therefore fail to fulfill its most important role, but it also contributes to spreading of the invaders within the host.

The specific molecular mechanism that allows *F. tularensis* to escape from the phagosome has not been completely elucidated. Previous reports have demonstrated that proteins encoded within the *Francisella* Pathogenicity Island (FPI) are important for *Francisella* phagosome escape and intracellular or intra-macrophage growth^8–14^. The majority of FPI genes encode components of an untypical type 6 secretion system (T6SS)^15^. Different bacteria exploit the secretion systems either to manipulate host cell defense mechanisms and/or to increase the parasite fitness. On a molecular level, they usually deliver effector proteins to the extracellular environment or directly into the host cell cytosol^16–19^. *Francisella tularensis* secretes effectors encompassing both the FPI proteins and *Francisella* virulence factors encoded outside of the FPI through its T6SS system^20–22^. We hypothesize that these proteins paralyze the host defense mechanisms by interacting with crucial molecular components thereof, and this results in transforming the adverse host milieu into a friendly niche for bacterial replication.

Proteins IglJ and VgrG belong to virulence factors of bacteria *F. tularensis*. Both proteins were identified as secreted by *Francisella tularensis* live vaccine strain (LVS)^20^ into macrophages and during *F. novicida* infection^21,22^. For *Francisella* virulence is essential its ability to escape from phagosomes and then replicate in macrophages, VgrG is required for this process in *F. novicida*^8^ and in LVS^9^, as well. Moreover, VgrG creates membrane-puncturing device and is one of the core component of T6SS^23^. IglJ protein was also found to be involved in intracellular growth of *F. novicida*^24^, and it is also important for an assembly of T6SS^25^. *F. tularensis* subsp. *tularensis* strain Schu S4 lacking the IglJ is defected in both phagosome escape and intracellular growth. Besides this, IglJ plays a critical role in the trafficking of *F. tularensis* to lysosomes^10^. So, the role of IglJ and VgrG is substantial in *Francisella* virulence despite the fact that their molecular mechanisms of pathogenesis are still unknown.

Recently, it was shown that effector protein of enterohemorrhagic *E.coli* KatN is translocated through T6SS into the host cell cytosol after the bacteria is phagocytized by macrophage. KatN has catalase activity and reduces the concentration of reactive oxygen species in host macrophages in order to facilitate its survival in the host cells^26^. *Vibrio cholerae* secretes the VgrG-1 protein that is capable to covalently cross-link the actin in host cell and leads to intestinal inflammation^27,28^. Bacteria like *Salmonella*, *Shigella*, and *Yersinia* use another secretion system—T3SS—to deliver their virulence factor into the host cell, where it manipulates the host cell cytoskeleton in order to facilitate the bacteria’s entry into the host cell^29–33^.

Proteomics offer many tools that can help to discover interactions between proteins from two organisms. Usually two such methods are linked together, such as cross-linking and liquid chromatography–mass spectrometry (LC–MS)^34^, affinity purification combined with LC–MS (AP-MS)^35^, or stable isotope labeling by amino acids in cell culture together with isobaric tags for relative and absolute quantitation (SILAC/iTRAQ) with LC-MS^36^. Although cross-linking allows identification of transient protein–protein interactions in vivo^37^, problems arise in trypsin digestion because most cross-linkers bind to the lysine residue and hinder the digestion^38^. Even the identification of low-abundance proteins and their interacting partners can be difficult in this type of experiment^39^. The strengths of AP-MS rest on posttranslational modification and multiprotein complex characterization in conditions corresponding to their physiological niche^40^. A weakness of this method lies in the huge quantities of proteins binding unspecifically to the matrix and creating the background. So, it is very difficult to distinguish real interactors with the bait proteins from their false counterparts^41^. SILAC-AP/MS provides a versatile, sensitive, specific, and accurate tool for identification of potential protein–protein interactions^42^. Its advantage is that it facilitates detection of low-abundance proteins^41^. The use of a negative control helps to screen out background proteins and increases confidence in the results. To improve the reliability of results, SILAC experiments can be swapped^43^.

The aim of this study was to identify host protein partners for two effectors encoded in FPI and known to be secreted via T6SS, VgrG and IglJ. To this end, we used an affinity purification approach based on SILAC that can distinguish true interaction protein pairs from nonspecific contaminants. We utilized a quantitative proteomic method consisting of tetracycline-inducible bait protein expression, SILAC, and affinity purification followed by mass spectrometry.

## Results

### Affinity purification combined with liquid chromatography–mass spectrometry (AP–MS)

**Figure 1:**
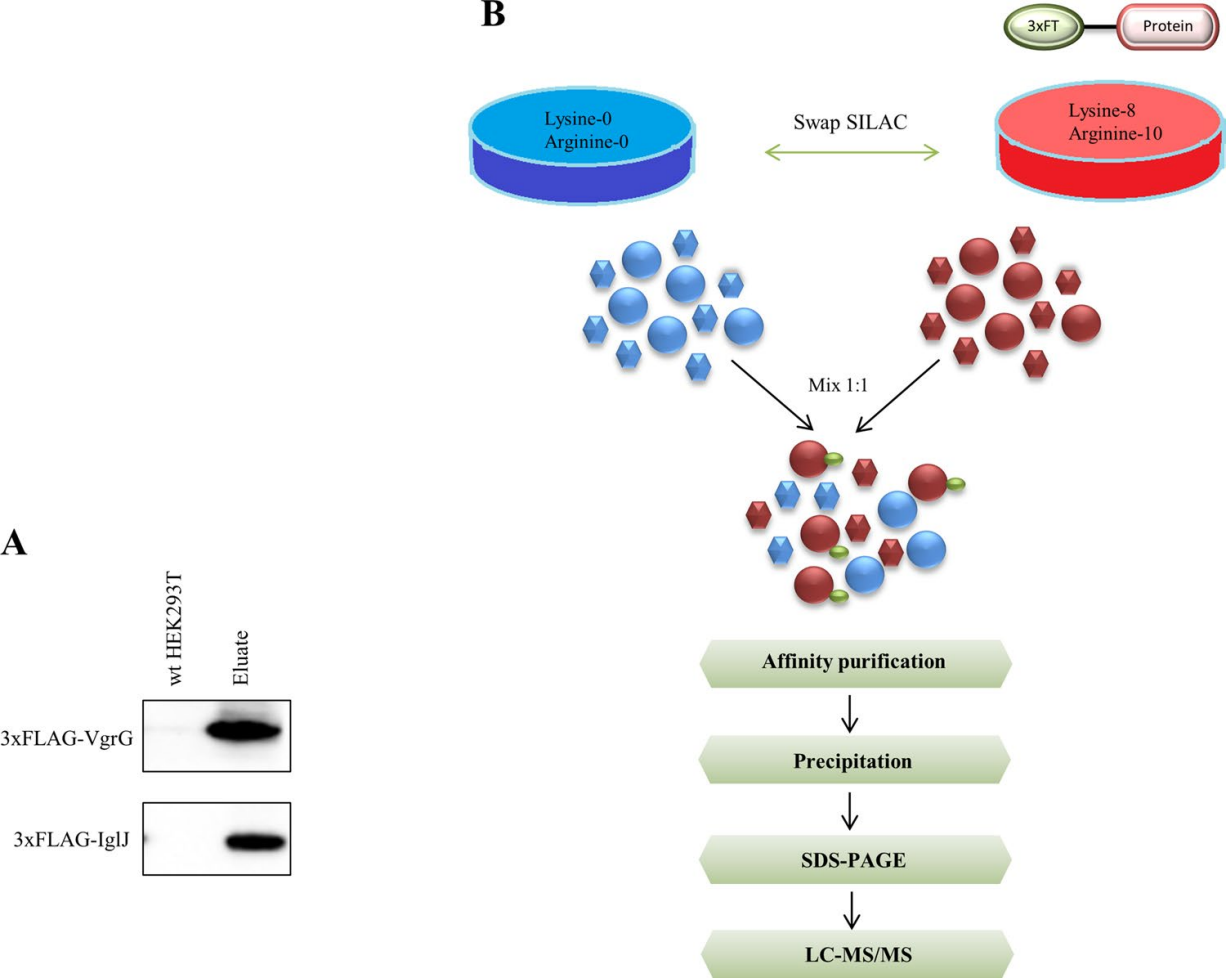
Overview of steps in identifying protein–protein interaction. (**A**) Detection of 3xFLAG-VgrG and 3xFLAG-IglJ protein in eluates and negative control by anti-FLAG antibody. (**B**) HEK 293 T cells stably expressing 3xFLAG-VgrG and 3xFLAG-IglJ were grown in “heavy” medium and control HEK293T cells were grown in “light” medium or vice versa in the swap SILAC experiment. The cell lysates containing equal amounts of protein were mixed 1:1. The protein complexes were purified using anti-FLAG M2 agarose beads. Eluates were precipitated, fractioned by SDS-PAGE, digested by trypsin, and then analyzed using LC–MS.

Western blot analysis was performed to determine whether the proteins 3xFLAG-VgrG and 3xFLAG-IglJ would be expressed in eukaryotic cells and would subsequently be present in eluates after affinity purification. This analysis confirmed VgrG 3xFLAG N-terminal tag and IglJ 3xFLAG N-terminal in eluate (Fig. 1A). Based on these results, the ensuing experiments were conducted and interacting partners were identified (Fig. 1B).

### Stable isotope labeling by amino acids in cell culture (SILAC) analysis

Overall, 577 distinct proteins were detected in all replicates of both samples. Totals of 76 proteins for VgrG effector and 60 proteins for IglJ effector were identified in 4 out of 6 biological replicates as potential interactors. All those proteins were subjected to strict filters. Furthermore, to filter out contaminants or nonspecific interactors, which are usually proteins with high affinity to agarose beads, we employed a negative control: HEK 293 T wild-type cells were subjected to the same procedure under the same conditions as was the real sample. Subsequently, we compared the proteins identified in the negative control with the set of proteins from the SILAC experiment, which helped us to exclude the nonspecific interactors from the initial list of identified proteins. After excluding of nonspecific interactors and detailed statistical and proteomic analysis we obtained five potential interaction proteins among the host cell for bacterial effector proteins (Table 1).

**Table 1:** List of potential interaction partner of VgrG and IglJ.

Effector	Potential interaction partner	log2 ratio H/L	Unique peptides
VgrG	CLIP-associating protein 1	2.5	30
	ATP-citrate synthase	1.9	24
	Exocyst complex component 4	1.4	16
	Ran GTPase-activating protein 1	1.4	27
	Exocyst complex component 2	1.1	9
IglJ	BAG family molecular chaperone regulator 2	2.4	7
	Prohibitin	2.2	9
	Angiomotin	2.2	44
	Prohibitin-2	1.9	15
	Apoptosis-inducing factor 1, mitochondrial	1.4	17

**Figure 2:**
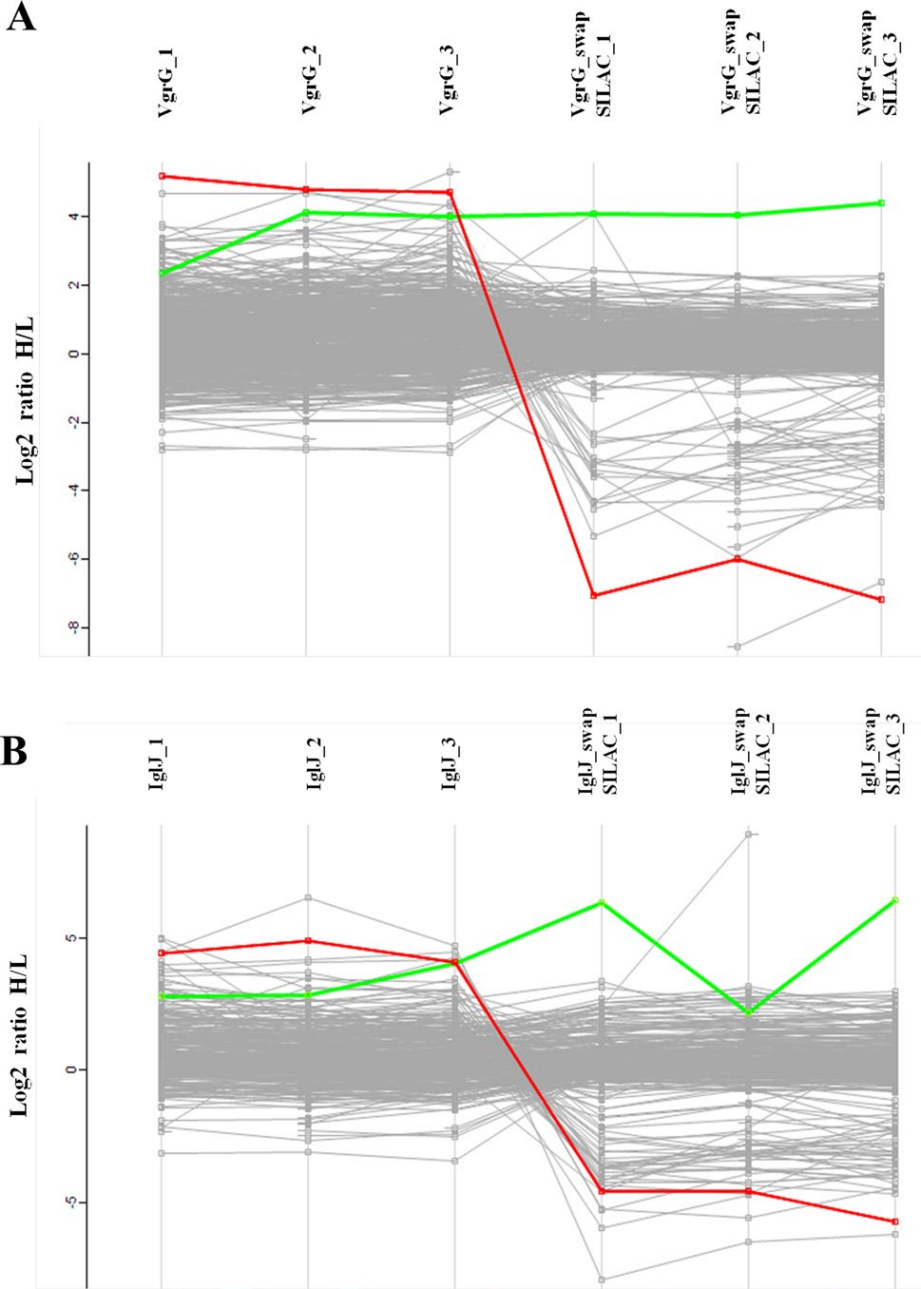
Profile plots. (**A**) Profile plot of sample HEK 293 T 3xFLAG-VgrG. Each line represents one identified potential interacting protein and its SILAC ratio (log2 scale) across all biological replicates. Green line represents fusion protein 3xFLAG VgrG. Red line represents common contaminant protein which is same in both profile plots. (**B**) Profile plot of sample HEK 293 T 3xFLAG-IglJ. Each line represents one identified potential interacting protein and its SILAC ratio (log2 scale) across all biological replicates. Green line represents fusion protein 3xFLAG IglJ. Red line represents common contaminant protein which is same in both profile plots.

Profile plot analysis showed that all bait proteins have the same SILAC ratio across all replicates (Fig. 2). We assume that interacting partners will have the same profile in all biological replicates. However, contaminants or background proteins present in sample have different SILAC ratios in the SILAC experiment and the swap SILAC experiment. The swap SILAC experiment also helped, therefore, to distinguish true interactors.

### Validation of interacting partners for VgrG and IglJ

**Figure 3:**
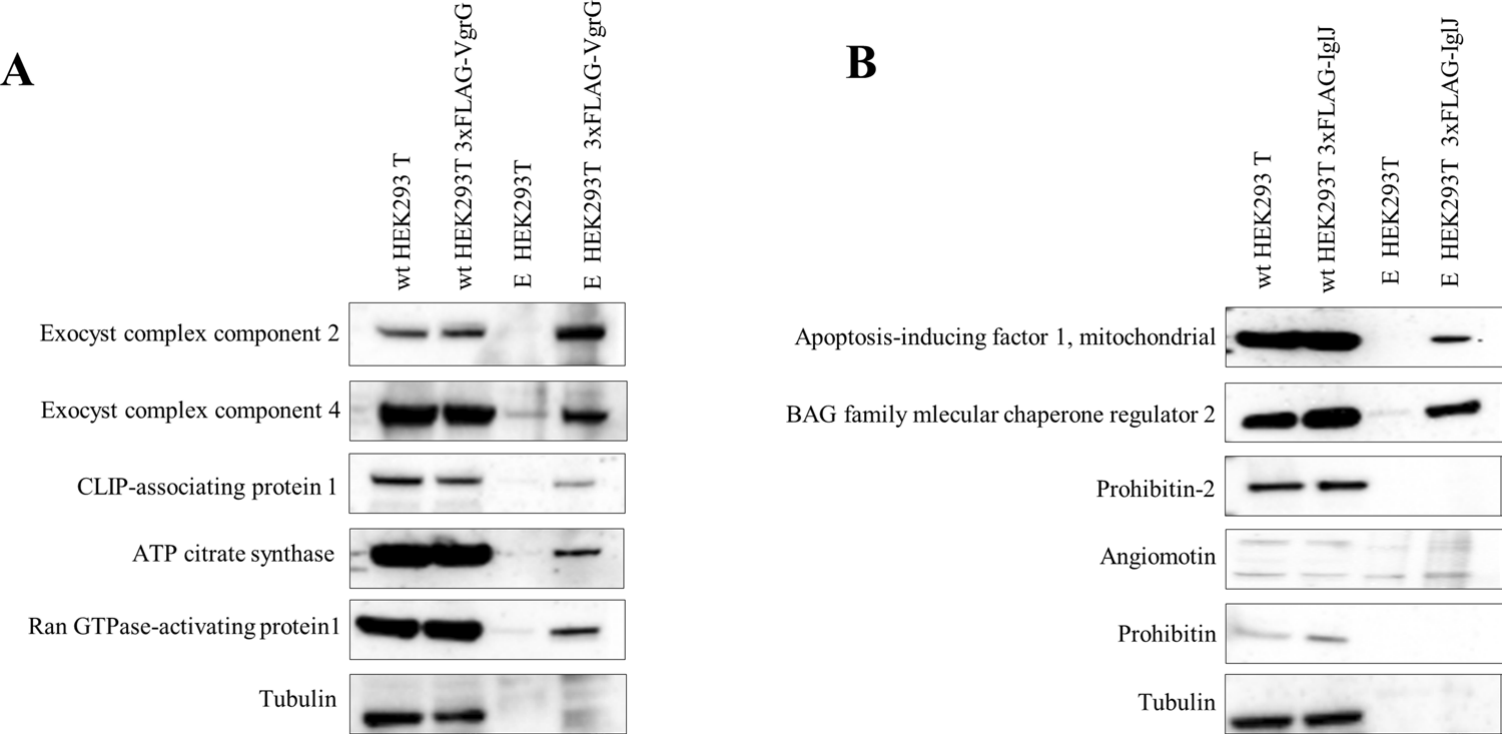
Validation of interacting partners for VgrG and IglJ proteins. Western blot analysis of the interacting partners for VgrG protein (**A**) and for IglJ protein (**B**). Lysates of wild-type cells and eluates were probed for immunoblotting analysis using specific antibodies for each identified interacting partner.

To confirm interaction partners for single bacterial effectors, we opted for affinity purification followed by western blot analysis. CLIP-associating protein 1, ATP-citrate synthase, Ran GTPase-activating protein 1 and exocyst complex component 2 were detected in eluate from the VgrG 3xFLAG-Tag expressing cell line. We consider these as interacting partners for bacterial effector protein VgrG (Fig. 3A). BAG family molecular chaperone regulator 2 and apoptosis-inducing factor 1, mitochondrial were present in eluates of HEK293T cells expressing bacterial effector 3xFLAG-IglJ protein (Fig. 3B). None of the previously identified interacting partners was detected in a negative control, thus confirming that they are not a part of the bead proteome^41^. Along with their interaction partners, 3xFLAG fusion proteins VgrG and IglJ were detected in the lysate of stably transfected HEK 293 T cells by western blot analysis using anti-FLAG antibody (data not shown).

## Discussion

A successful intracellular pathogen needs to overcome host defense mechanisms, evade immune surveillance, and create a friendly niche for its replication. Mitochondria are cellular organelles regulating various metabolic pathways, immune signaling, and apoptosis^44,45^. It is not surprising, therefore, that a number of bacterial virulence factors attack mitochondria very early after intracellular invasion^33^. Apoptosis inducing factor (AIF), a flavoprotein located in intermembrane space of mitochondria, was found among proteins interacting with IglJ^34^. This protein is capable to dissipate the mitochondrial transmembrane potential and release the apoptogenic protein cytochrome C. AIF also induces translocation of the phosphatidylserine on the plasma membrane and activates the condensation of nuclear chromatine^46^. The interaction between IglJ and AIF may impact upon the intrinsic pathway of apoptosis. Recently, the impaired process for activation of caspase-3, caspase-8, and caspase-9, as well as significantly impaired apoptosis triggered by Fas crosslinking, was described in polymorphonuclear leukocytes infected by *F. tularensis* LVS. The inhibition of apoptosis was accompanied by sustained mitochondrial integrity and preserved the mitochondrial membrane potential^47,48^. Moreover, *F. tularensis* subsp. *tularensis* improves mitochondrial bioenergetics at the beginning of the infection process in macrophages in order to inhibit apoptosis and allow the bacteria’s replication^49^. *Francisella tularensis* is not the only intracellular bacterium capable of manipulating host mitochondria. *Mycobacterium tuberculosis* inhibits apoptosis in infected macrophages through upregulation of anti-apoptotic proteins, such as B-cell lymphoma (Bcl)-2 and Mcl-1, and by depletion of cytochrome C^50^. Similarly, *Legionella pneumophila* is able to prevent apoptosis in host macrophages by secreting bacterial effectors SdhA and SidF. SidF specifically interacts with proteins from the Bcl-2 family BNIP3 and Bcl-rambo^51,52^. This bacteria has a protein in its genome—known as mitochondrial fragmentation factor (MitF)—for inducing mitochondrial fragmentation upon infection^53^. Infection-induced apoptosis of host epithelial cells is prevented by *Salmonella typhimurium* and its secreted effector protein SopB (*Salmonella* outer protein B). The mechanism works by inhibiting the production of mitochondrial superoxide ROS (mROS) through binding to the cytosolic tumor necrosis factor receptor associated factor 6 (TRAF6)^54^. Also, fimbrial protein subunit A (FimA) of *Salmonella* targets outer mitochondrial membrane protein VDAC1 that disables release of cytochrome C^55^. Therefore, controlling apoptosis or mitochondria functions could be a unique strategy of bacteria to limit inflammation and control cell death.

The exocyst is a hetero-octameric complex containing eight subunits: Sec3, Sec5, Sec6, Sec8, Sec10, Sec15, Exo70, and Exo84. Functions of this complex include exocytosis, cytokinesis, cell migration and growth, tumorigenesis, and primary ciliogenesis. It also mediates connection between secretory vesicles and target membrane in order to promote SNARE complex formation^56–58^. Some components of the exocyst complex are involved in autophagy^59,60^. Mutants in Sec3, Sec5, Sec6, Sec8, and Sec 10 impair Atg9 trafficking, thereby leading to decrease in delivery of membrane to the site of autophagosome biogenesis^61^. *Legionella pneumophila* subverts the tethering functions of the exocyst components to promote the fusion of endoplasmic reticulum-derived vesicles with the *Legionella*- containing vacuole. This creates for the pathogen a specialized compartment that supports bacterial replication^62^. Intracellular bacteria *Listeria monocytogenes* and *S. typhimurium* use the exocyst for invading the host cell. In *Listeria*, bacterial surface protein InlB promotes entry by stimulating exocytosis through recruitment of Exo70^63^. Exocyst complex components (Sec5, Sec8) are present at sites of *Salmonella* invasion. They lead to membrane ruffling and macropinocytosis entry of attached bacteria. The *Salmonella* effector protein SipC directly interacts with actin and Exo70. Knockdown of Sec5 reduces membrane ruffling and bacterial invasion^64^. The exocyst is involved in phagosome maturation in human endothelial cells infected by *Staphylococcus aureus*. Knockdown of exocyst proteins Sec8 and Exo70 significantly reduce Lamp1 positive phagolysosomes and association of Rab11 with endothelial cell phagosomes. Therefore, interaction of the VgrG protein with the exocyst components might be the first step in blocking maturation of the *Francisella-*containing phagosome. Experiments to confirm this assumption are currently under preparation.

The interaction of the VgrG protein with ATP-citrate synthase (ACLY) can affect the generation of cytosolic acetyl-CoA in the cell. Acetyl-CoA is a central molecule in signaling, epigenetics, cell metabolism, and synthesis of fatty acids, UDP-N-acetylglucosamine, and cholesterol. It is required for protein acetylation and also is important in histone acetylation^65,66^. In macrophages, inhibition of ACLY activity or gene silencing has been shown to cause reduction in NO and ROS levels^67^. Depletion of nucleo-cytosolic acetyl-CoA stimulates autophagy. Once proteins of the autophagic machinery are acetylated, the process of autophagy is inhibited. On the other hand, a decrease in the acetyl-CoA/CoA ratio could promote cell survival, because the apoptotic activity of CASP2 may rely on N^α^-acetylation^66^.

In this study, we explored protein–protein interactions between some *Francisella* effector proteins and their host cell counterparts. Our data indicate that VgrG and the IglJ may play a role in controlling phagosome maturation and the mitochondrial pathway of apoptosis, respectively. Because the biological roles of the proteins encoded within the FPI are currently unknown, these discoveries suggest new directions for future investigation into the molecular mechanism of *Francisella* virulence.

## Methods

### Plasmid construction and stable transfection

HEK 293 T cells (ATCC-CRL-3216) were obtained from Cellulonet (Lyon, France), tested to be mycoplasma-free, and grown in Dulbecco’s modified Eagle’s medium (DMEM) with glutaMAX-I and supplemented with 10% fetal bovine serum, 100 IU/μl penicillin, and 100 μg/μl streptomycin (all from Thermo Fisher Scientific, MA, USA). Sequences of *iglJ* and *vgrG* genes of *Francisella novicida* were codon-optimized for human expression (Genewiz, Leipzig, Germany). IglJ and VgrG were cloned in-frame with a 3xFLAG N-terminal tag into the GFP-expressing plasmid pINDUCER21 under the control of a doxycycline-inducible promoter through the pENTR1A (Invitrogen, MA, USA) vector using NotI and XhoI enzymes. To obtain, stable cell lines, HEK 293 T cells were transduced using lentiviral particles. Briefly, lentiviral particles were produced in 293 T cells using pMD2.G and psPAX2 (Addgene, MA, USA), as well as pINDUCER-21 plasmids expressing 3xFLAG-VgrG and 3xFLAG-IglJ proteins. Lentiviruses were concentrated by ultracentrifugation on a sucrose gradient and used to transduce HEK 293 T cells by spinoculation. At day 7 post-transduction, HEK 293 T cells having stably integrated the lentiviruses were sorted based upon GFP expression on an Aria cell sorter (BD Biosciences, CA, USA). Protein expression was induced by treatment with 1 μg/μl doxycycline for 16 h before preparation of cell lysate.

### Stable isotope labelling

The HEK 293 T cells stably transfected with pINDUCER-21 plasmids expressing 3xFLAG-VgrG and 3xFLAG-IglJ proteins were grown in “heavy” SILAC DMEM without lysine and arginine (Thermo Fisher Scientific), supplemented with 10% dialyzed fetal bovine serum (Sigma-Aldrich, MO, USA), 84 mg/l ^13^C_6_^15^N_4_, L-arginine-HCl, 146 mg/l, and ^13^C_6_^15^N_2_ L-lysine-HCl. The cells were grown in 300 mg/l concentration of L-proline (all from Sigma-Aldrich) to prevent arginine-to-proline conversion. At the same time, wild-type HEK 293 T cells were grown in “light” DMEM (Thermo Fisher Scientific), supplemented with 10% fetal bovine serum (Sigma-Aldrich). Medium was changed every 2 or 3 days for at least five cell divisions. A swap SILAC experiment was conducted while reversing treatments, with wild-type HEK293T cells grown in “heavy” SILAC DMEM and stably transfected cell line grown in “light” DMEM.

### Affinity purification and sample preparation for liquid chromatography–mass spectrometry (LC–MS/MS)

Cells grown in the “heavy” and “light” media were harvested and lysed in lysis buffer (50 mM Tris–HCl, pH 7.4; 150 mM NaCl; 1 mM EDTA; 7.5% glycerol; 1% Triton X-100) supplemented with EDTA-free protease inhibitors (Roche, Switzerland). Benzonase was added to minimize nucleic acid contamination. The protein concentration of samples was determined and light and heavy lysates were mixed in a 1:1 ratio. Lysate was incubated with the anti-FLAG M2 affinity gel (Sigma-Aldrich) at 4 °C overnight. The resin was washed with the lysis buffer to remove all nonspecific proteins. The bound FLAG-tag protein along with its interaction partners was eluted from the column with a solution containing FLAG peptide. Eluates were further precipitated by sodium deoxycholate, trichloroacetic acid (TCA), and acetone to remove salts and detergents. Pellets were dissolved in LDS buffer and separated by SDS-PAGE (Invitrogen). Gels after electrophoresis were stained with colloidal blue (Invitrogen) and cut into 10 fractions. In-gel trypsin digestion of the excised protein spots was done. Prior to nanoLC-MS analysis, the samples were desalted using custom-made reversed-phase microcolumns. Peptides were eluted using nonlinear gradient with gradually increasing acetonitrile (ACN) content in the range 2–40% (v/v) with 0.1% trifluoroacetic acid (TFA) from the desalting microcolumn^68^. (Fig. 1B).

### LC–MS/MS analysis and database searching

Peptides from the complex mixtures were first dissolved in 20 µl of 2% ACN/0.1% TFA and 1 µl was analyzed using the UltiMate 3,000 HPLC system (Dionex, CA, USA), consisting of a µ-precolumn (300 µm × 5 mm, PepMap C18, 5 µm, 100 Å; Dionex) connected to the analytical NanoEase column (100 µm × 150 mm, Atlantis C18, 3 µm, 100 Å; Waters, Milford, MA, USA). The separation was performed with linear gradient of 5–45% ACN/0.1% TFA over 25 min under a flow rate of 360 nl/min and UV detection set to 215 nm. The separation of peptides for nanoLC-MS/MS analysis was done using the UltiMate 3,000 RSLC-nano HPLC system (Dionex) with a trap column (75 µm × 20 mm) packed with 3 µm Acclaim PepMap100 C18 particles and a separation column (75 µm × 150 mm) packed with 2 µm Acclaim PepMap RSLC C18 particles. The separation was performed with linear gradient using 3–44% ACN over 47 min under a flow rate of 300 nl/min and analyzed with the Q Exactive system (Thermo Fisher Scientific) in positive mode with full MS scan (350–1,650 m/z) at 70,000 full width at half maximum (FWHM) and with top 10 precursors in MS/MS at 17,500 FWHM.

MaxQuant software (ver. 1.6.1.0) was used for protein identification of MS/MS spectra^69^. The data were searched against the FASTA database consisting of reference proteomes of *Homo sapiens* downloaded from Uniprot (UP000005640; 5 July 2018) and *Francisella tularensis* subsp. *novicida* (strain U112) downloaded from Uniprot (UP000000762; 5 July 2018). Parameters of MaxQuant search were: mass tolerance for the first search 20 ppm, for the second search from recalibrated spectra 4.5 ppm; minimal length of peptide 7 amino acids and maximal mass of peptide 4,600 Da. For peptide quantitation, Arg8 and Lys10 were set as labels in the heavy channel with re-quantify function enabled. Trypsin with 2 missed cleavages was set as a protease. Oxidation of methionine and acetylation of protein N-terminus were set as variable modifications. Mass tolerance for fragments in MS/MS was 20 ppm.

All statistical and proteomic analysis and were done in the program Perseus (ver.1.6.2.3)^70^. Data were log2 transformed, then filtered based on SILAC (light/heavy) ratio (L/H ratio > 2 (Log_2_ L/H ratio > 1)). Finally, to assess statistical significance, a two-tailed *t*-test was performed using the permutation-based false discovery rate, with a cutoff at 0.05. To combine the SILAC and swap SILAC runs, the data were filtered for having counted SILAC ratio in at least two out of three replicates of one experimental group.

### Affinity purification and immunoblotting

Cells were lysed in lysis buffer (50 mM Tris–HCl, pH 7.4; 150 mM NaCl; 1 mM EDTA; 7.5% glycerol; 1% Triton X-100) supplemented with EDTA-free protease inhibitors (Roche). Benzonase was added to minimize nucleic acid contamination. The protein concentration of samples was determined and affinity purification was performed. Eluates were precipitated by sodium deoxycholate, trichloroacetic acid, and acetone. Equivalent protein quantities of lysates and pellets after precipitation were subjected to SDS-PAGE and transferred to nitrocellulose membranes. Membranes were blocked using 5% dry milk in tris-buffered saline containing 0.05% Tween-20. Membranes were then probed with selected primary antibodies, followed by the appropriate HRP-conjugated secondary antibodies (Dako, CA, USA). The following monoclonal primary antibodies were used: anti-FLAG-tag (Sigma-Aldrich), anti-BAG2 (Abcam, UK), anti-ATP citrate (Abcam), anti-RANGAP1 (Abcam), antiCLASP-1 (Abcam), anti-Sec5 (Santa Cruz Biotechnology, CA, USA), anti-Sec8 (Biocompare, CA, USA), anti-Prohibitin 2 (Santa Cruz Biotechnology), anti-Angiomotin (Santa Cruz Biotechnology, USA), anti AIF (Abcam), and anti-Prohibitin (Abcam).

## Supplementary information

Supplementary Figures
