# Supplementary material for: Using proteomics to identify host cell interaction partners for VgrG and IglJ
Source: Sci Rep. 2020 Sep 3;10:14612. doi: 10.1038/s41598-020-71641-3 (PMC7471685; doi:10.1038/s41598-020-71641-3)

Supplementary information

Using proteomics to identify host cell interaction partners for VgrG and IglJ

Magdalena Proksova[1], Helena Rehulkova[1], Pavel Rehulka[1], Claire Lays[2], Juraj Lenco[3], Jiri Stulik[1*]

[1] *Department of Molecular Pathology and Biology, Faculty of Military Health Sciences, University of Defence, Hradec Kralove, Czech Republic*

[2] *CIRI, International Center for Infectiology Research, Inserm U1111, CNRS, UMR5308, Lyon, France*

[3] *Faculty of Pharmacy, Charles University, Hradec Kralove, Czech Republic*

Correspondence: Jiri Stulik

University of Defence, Faculty of Military Health Sciences

Department of Molecular Pathology and Biology

Trebesska 1575

Hradec Kralove

Czech Republic

Email: jiri.stulik@unob.cz

Tel.: +420 495 973 253220

Supplementary Figure S1 3
Supplementary Figure S2 4
Supplementary Figure S3 7

Supplementary Figure S1

Uncropped membranes from images in figure 1B. Red boxes indicate lanes included in figure in manuscript. Proteins were detected by AntiFlag antibody. Pictures were obtain by iBright™ FL1500 Imaging System (Thermo Fisher Scientific).

3xFLAG-VgrG

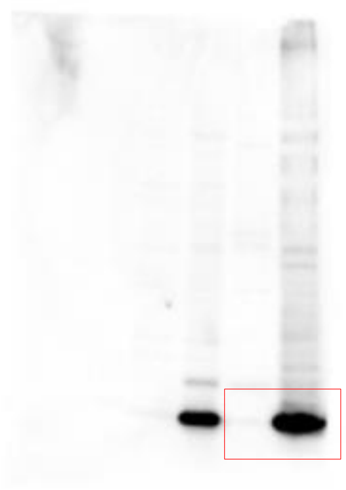

3xFLAG-IglJ

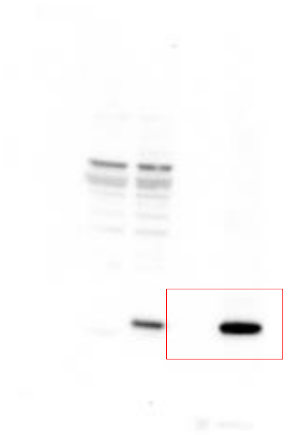

Supplementary Figure S2

Uncropped membranes from images in figure 3A. Red boxes indicate lanes included in figure in manuscript. Proteins were detected by antibodies against Exocyst complex component 2, Exocyst complex component 4, CLIP-associating protein 1, ATP citrate synthase, Ran GTPase activating protein 1 and Tubulin. Pictures were obtain by iBright™ FL1500 Imaging System (Thermo Fisher Scientific).

Exocyst complex component 2

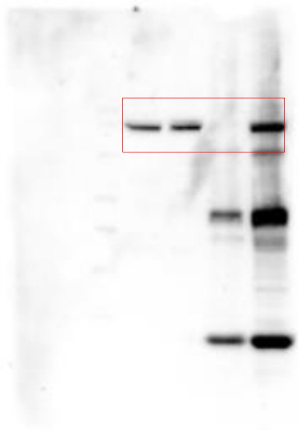

Exocyst complex component 4

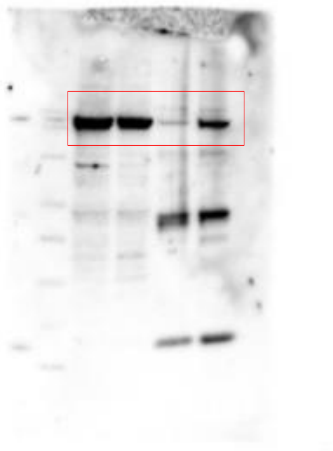

CLIP-associating protein 1

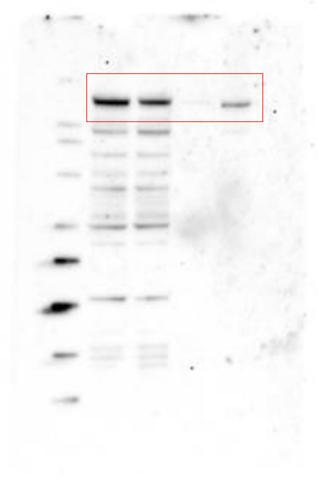

ATP citrate synthase

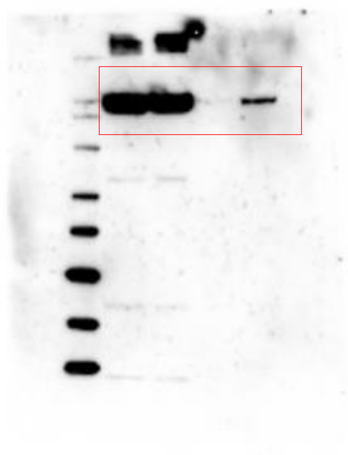

Ran GTPase activating protein 1

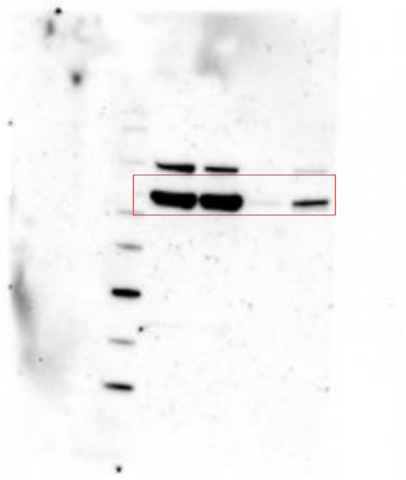

Tubulin

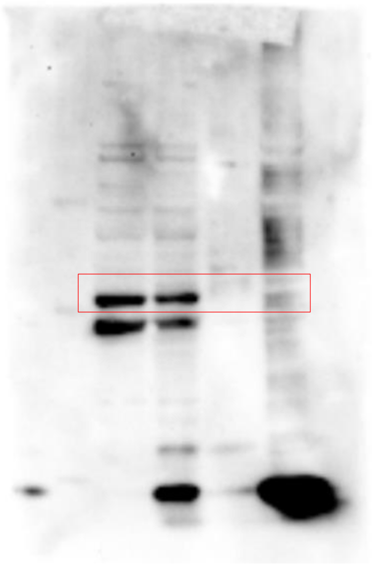

Supplementary Figure S3

Uncropped membranes from images in figure 3B. Red boxes indicate lanes included in figure in manuscript. Proteins were detected by antibodies against Apoptosis-inducing factor 1, mitochondrial, BAG family molecular chaperone regulator 2, Prohibitin-2, Angiomotin, Prohibitin and Tubulin. Pictures were obtain by iBright™ FL1500 Imaging System (Thermo Fisher Scientific).

Apoptosis-inducing factor 1, mitochondrial

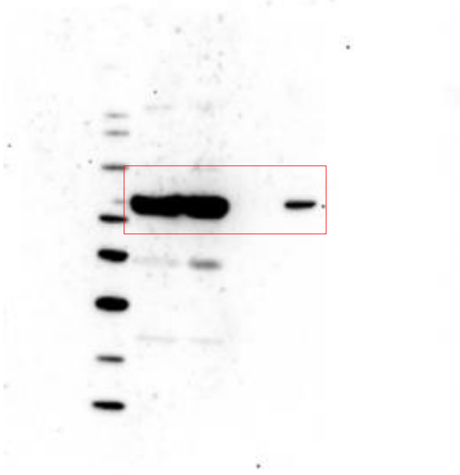

BAG family molecular chaperone regulator 2

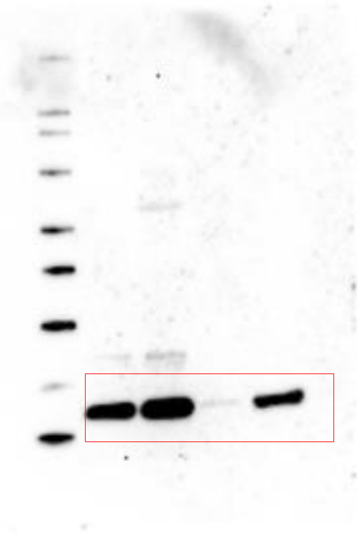

Prohibitin-2

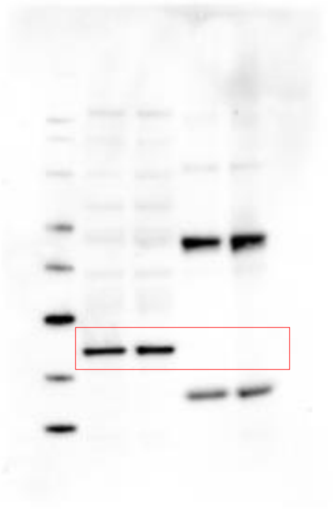

Angiomotin

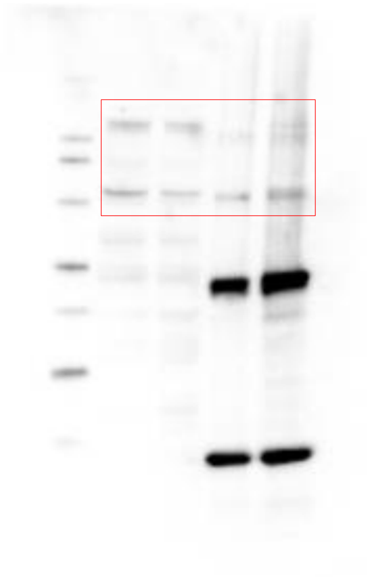

Prohibitin

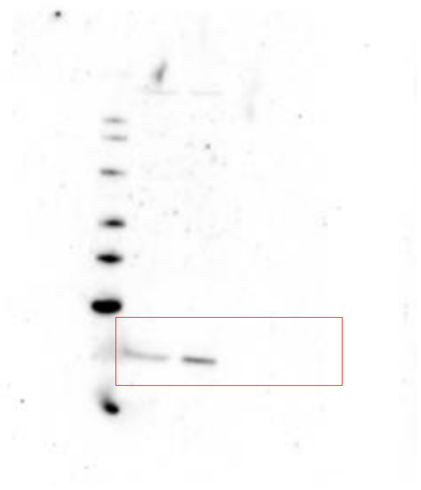

Tubulin

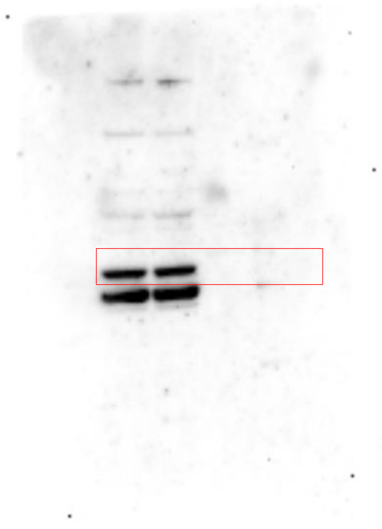

Supplement: Supplementary file 1 — Supplementary Figures [file 41598_2020_71641_MOESM1_ESM.pdf]
